# Direct aortic Y-graft for cardiopulmonary bypass initiation and Impella 5.5 insertion through a single aortotomy

**DOI:** 10.1016/j.xjtc.2026.102444

**Published:** 2026-05-25

**Authors:** Tommaso Cambiaghi

**Affiliations:** Cardiothoracic and Vascular Surgery Division, Monument Health Heart and Vascular Institute, Rapid City, SD

**Figure:**
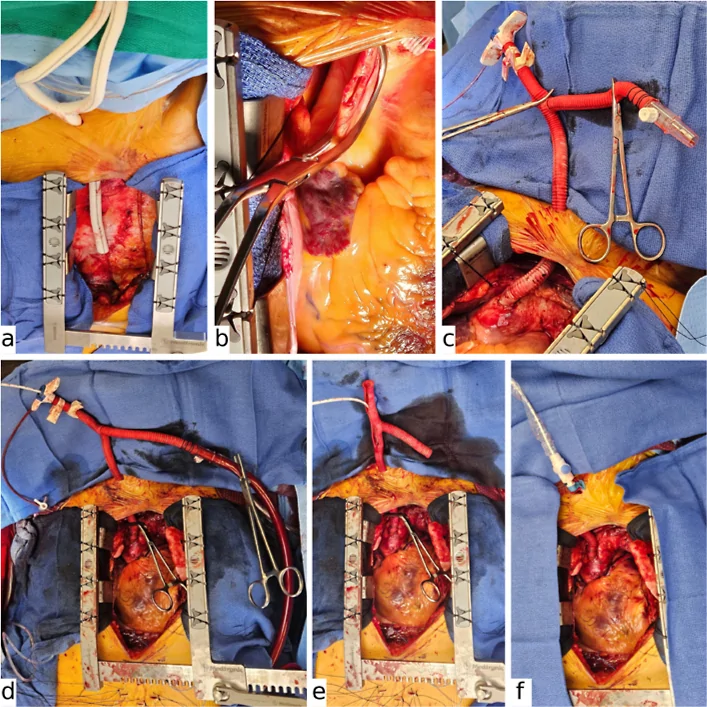
Step-by-step placement and removal of Y-graft for Impella and cardiopulmonary bypass.

**Central Message**

Direct aortic Y-graft allows cardiopulmonary bypass initiation and insertion of Impella 5.5 reducing the number of aortotomies and streamlining the initiation of mechanical circulatory support.

This report describes a variation of an established clinical approach; therefore, it did not constitute human subjects research and did not require institutional review board approval or consent. No patient-specific data are included in the manuscript.

When prepping and draping the patient, care should be taken to include the base of the neck in the surgical field so that then the graft can be tunneled between the heads of the sternocleidomastoid muscle. Before the procedure is initiated, a 10-mm woven Dacron graft is cut and sewn into a Y configuration using a 5-0 PROLENE suture (Ethicon) and the aid of a vascular sealant (Figure 1). An alternative is to use a premade T-shaped 10-mm knitted Dacron graft, usually reserved for axillobifemoral bypasses, and with greater material porosity that can lead to unnecessary blood loss.

**Figure 1:**
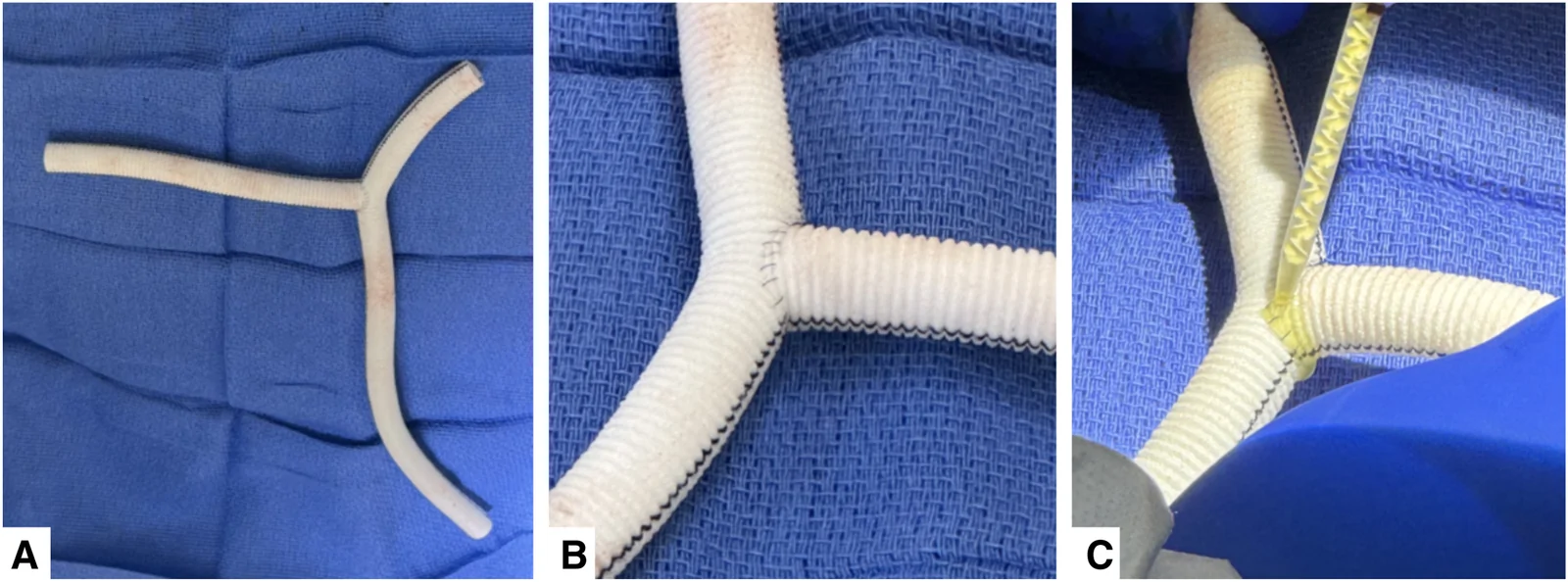
Y Dacron creation (A) starting from a 10-mm woven Dacron straight graft cut and (B) sewn with a 5-0 PROLENE suture and (C) sealed with Baxter PREVELEAK.

After median sternotomy, the operation is set up in routine fashion up until the point of cannulation. At this point, a 10-mm incision is made at the base of the right neck, a tunnel to the mediastinum is created bluntly, a 10-mm Y Dacron graft is passed into the tunnel with both limbs of the Y sitting outside the chest cavity (Figure 2, *A*).

**Figure 2:**
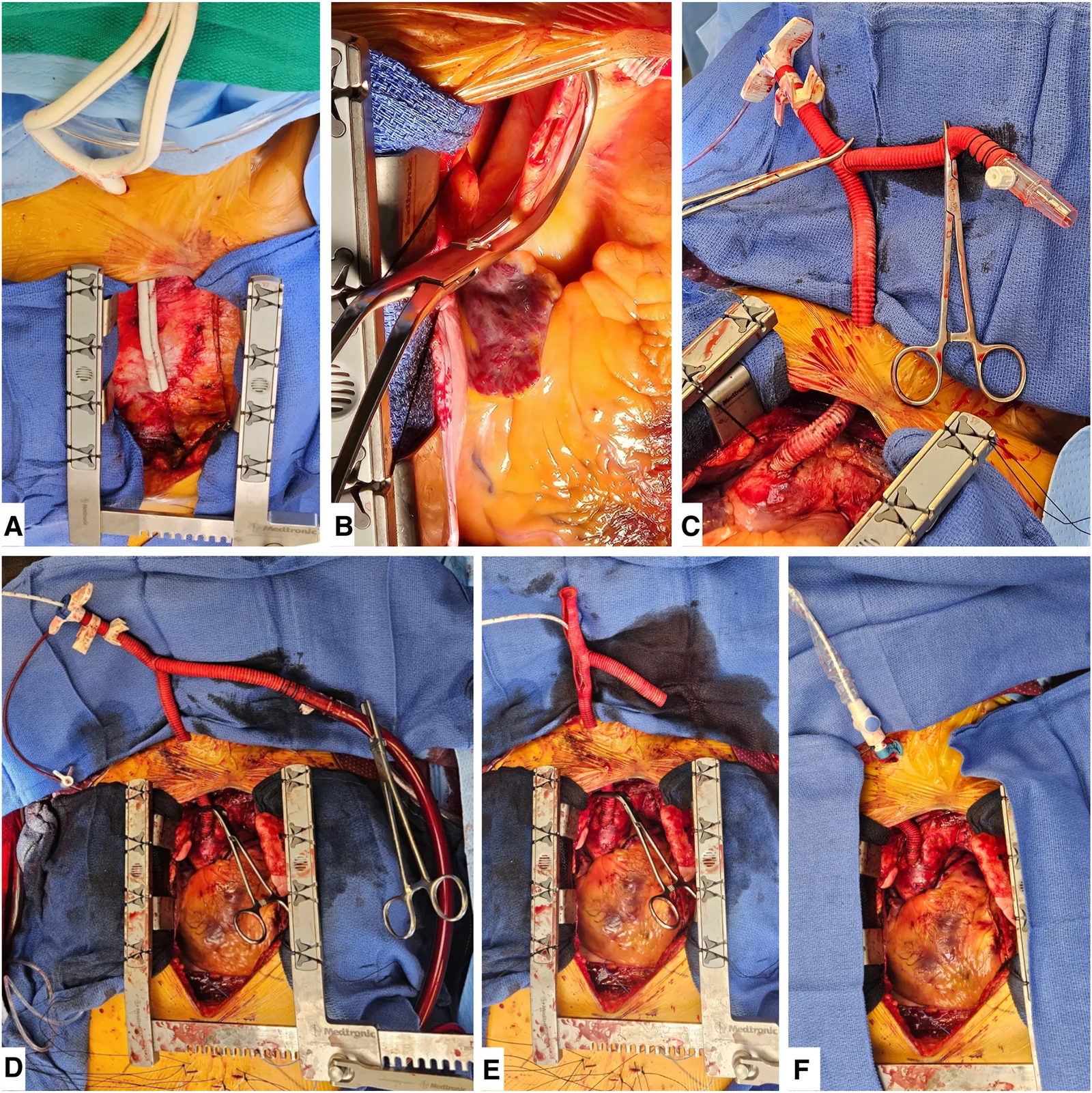
The Y-graft is tunneled from the neck through a separate stab incision between the heads of the sternocleidomastoid muscle (A). An aortic partial clamp is applied, a 10-mm aortotomy is created (B), and graft anastomosis is completed. The graft is then deaired through the Impella peel-away sheath and a perfusion adaptor (C). Before the patient is weaned from cardiopulmonary bypass, the Impella is placed in standard fashion (D) and then the graft and arterial line are clamped and the graft is cut past its bifurcation (E). The graft is then shortened and the device secured to the skin with enough intrathoracic redundancy to allow for safe removal (F). The patient has provided consent for the use of these photographs.

After systemic heparinization is established, instead of cannulating the aorta directly, a side-biting clamp is placed at the most cranial portion of the ascending aorta that can be controlled without impairing hemodynamics or flow through the supra-aortic trunks. An aortotomy is performed and enlarged with a punch cutter to at least 10 mm of diameter (Figure 2, *B*). Care must be taken in ensuring the aortotomy site is at least 7 cm above the aortic annulus to allow for the outlet of the Impella 5.5 to sit in the ascending aorta and not in the Dacron conduit once placed across the valve.

Once the aortic anastomosis is completed, the Impella peel-away insertion sheath is placed on one limb of the Y graft, a perfusion adaptor is placed on the other limb (Figure 2, *C*), and both are deaired. The perfusion adaptor is then connected to the arterial limb of the cardiopulmonary bypass (CPB) circuit.

The procedure can then be carried out in standard fashion until the moment of separation from CPB. Before starting to wean the patient off CPB, the wire and catheter of choice (at our center, a BER catheter with a 0.035-inch Newton wire then exchanged for the 0.018-inch wire included in the Impella 5.5 kit) are inserted through the Impella peel-away sheath, and the aortic valve is crossed under transesophageal echocardiography or fluoroscopic guidance. The Impella 5.5 device is inserted into the left ventricle over the wire, depth is confirmed with transesophageal echocardiography, and the wire is removed before the initiation of temporary mechanical circulatory support.

As the heart is allowed to fill and eject, the Impella settings are increased to P7 and the CPB is weaned. Once the patient is hemodynamically stable and any residual volume is reintroduced from the CPB reservoir, the Dacron conduit is clamped with a padded clamp (eg, Fogarty Hydrogrip) and the arterial tubing is clamped (Figure 2, *D*). The peel-away sheath is then removed and the Y-graft cut longitudinally until proximal to the bifurcation point (Figure 2, *E*), where the straight portion of the graft is then tied around the Impella and the clamp is removed. Some redundancy is allowed to ensure safe removal of the Impella device and ligation of the graft once clinically indicated, and the device is secured at the skin at the base of the right neck (Figure 2, *F*). The remainder of the closure is performed in standard fashion with a repeat Impella 5.5 depth check after sternal approximation.

## Discussion

Direct aortic cannulation for Impella 5.5 placement is a valid option for patients undergoing cardiac surgery and planned temporary mechanical circulatory support. It avoids the need for separate axillary exposure and the risks of limb malperfusion and chest wall hematoma.

The downside of central insertion is that it requires a separate aortotomy, at least 7 cm from the aortic annulus, which often limits the available options for site of aortic cannula insertion and can make the aortic decannulation process more cumbersome because of proximity with the Impella conduit. Furthermore, creation of the Impella conduit after aortic crossclamping extends the myocardial ischemic time and the overall pump time.

By using this technique, the number of aortotomies is reduced and potentially the consequent risk of bleeding is reduced, the decannulation process is simplified and controlled, and the myocardial ischemic time is not extended.

Simultaneous Impella and extracorporeal circulation through a single graft has been described in an axillary setup for ECPella (ie, combined configuration of venoarterial extracorporeal membrane oxygenation and the Impella) but never in a central configuration.^1^ The downsides of this technique are that it requires partial clamping of the aorta, with its associated risks,^2^ and that it leaves the Impella conduit distal to the aortic crossclamp, therefore preventing crossing of the aortic valve with the device under direct vision in procedures like aortic valve replacement. In these cases, a separate aortic cannulation site and aortic conduit below the clamp site would be preferred. Finally, having the Impella driveline indwelling in the CPB arterial line could potentially cause partial obstruction of flow during manipulations, although this has not proved to be a limiting factor in our experience.

## Conclusions

The direct aortic Y-graft allows CPB initiation and insertion of the Impella 5.5, reducing the number of aortotomies and streamlining the initiation of mechanical circulatory support.

## Conflict of Interest Statement

The author reported no conflicts of interest.

The *Journal* policy requires editors and reviewers to disclose conflicts of interest and to decline handling or reviewing manuscripts for which they may have a conflict of interest. The editors and reviewers of this article have no conflicts of interest.
